# Prevalence of Alcohol and Tobacco Use in India and Implications for COVID-19 - Niyantrita Madhumeha Bharata Study Projections

**DOI:** 10.25122/jml-2020-0079

**Published:** 2020

**Authors:** Madhava Sai Sivapuram, Raghuram Nagarathna, Akshay Anand, Suchitra Patil, Amit Singh, Hongasandra Ramarao Nagendra

**Affiliations:** 1. Department of General Medicine, Dr. Pinnamaneni Siddhartha Institute of Medical Sciences and Research Foundation, Chinna-Avutapalli, Andhra Pradesh, India; 2. Department of Life Sciences, Swami Vivekananda Yoga Anusandhana Samsthana, Bengaluru, India; 3. Neuroscience Research Lab, Department of Neurology, Postgraduate Institute of Medical Education and Research, Chandigarh, India

**Keywords:** Legal substance abuse, tobacco, alcohol, Niyantrita Madhumeha Bharata, diabetic yoga protocol

## Abstract

Abuse of legal substances in India includes alcohol and tobacco, which are the major risk factors for various non-communicable diseases and deaths. The current pandemic has identified tobacco consumption as a risk factor for COVID-19, highlighting the need to control substance abuse. The objective of this study was to estimate the prevalence of substance abuse in India and discuss the cost-effective public health strategies (such as yoga) to alleviate COVID-related anxiety in order to prevent substance abuse and its associated co-morbidities such as type 2 diabetes mellitus. This study reports the data on tobacco and alcohol abuse from a nationwide randomized two-arm diabetes control trial (Niyantrita Madhumeha Bharata, 2017) conducted by the Indian Yoga Association (IYA) through Swami Vivekananda Yoga Anusandhana Samsthana (S-VYASA), Bengaluru. Data of 30,354 participants who abuse tobacco and 30,159 participants who abuse alcohol were collected all over India. The prevalence is estimated at around 8.7% for alcohol abuse and 7.9% for tobacco abuse, Arunachal Pradesh state ranking the highest regarding both alcohol and tobacco abuse, while the Tripura state ranked the lowest. School and college-based mandatory yoga programs need to be implemented to prevent the increase of substance abuse in India to alleviate the psychosocial stress of adolescents and college-going students, besides the installation of the mindfulness-based diabetes yoga protocol (DYP) in the wellness centers of Ayushman Bharat.

## Introduction

Alcohol and tobacco are legal substances that are often abused in India and constitute major risk factors for various diseases, also increasing the burden of non-communicable diseases, especially when these substances are used by the general public [1, 2].

Globally, 1.3 billion people are using tobacco products, and the annual death rate is around six million [3]. According to the 2018 World Health Organization (WHO) factsheet, tobacco abuse and addiction kill more than one million people in India, which is one-sixth of the world deaths due to tobacco usage and accounts for 9.5% of all deaths in India [4]. These facts inform us of the dangers of tobacco consumption on one’s general health. Furthermore, the WHO reports indicate that tobacco-related deaths will rise to a million, accounting for 10% of global deaths by 2030, if appropriate measures are not taken [5]. Tobacco is a plant that is grown, and the leaves of the tobacco plant are dried and further fermented. The fermented tobacco leaves are converted to tobacco products, which can be either smoked as tobacco products or used as smokeless tobacco products. The smoked tobacco products include cigarettes, cigars, bidis, rolled cigarettes, cheroots, hookah pipes, tobacco rolled in maize leaf and newspaper, chillum [6, 7], while the smokeless tobacco products available include khaini, betel quid with tobacco, gutka, tobacco lime mixture, pan masala, oral tobacco, snuff and others [7].

Currently, electronic cigarettes known as e-cigarettes and flavored tobacco products are on the rise in India and across the world [8]. The chief ingredient of tobacco causing addiction is nicotine, which is a carcinogenic agent responsible for various diseases and has high mortality [9]. Tobacco consumption leads to multiple diseases such as ischemic heart disease, hypertension, neoplasia, especially lung cancer, throat cancer, tracheal cancer, oral cancer, oesophageal cancers, chronic obstructive pulmonary disease (COPD), lower respiratory tract infections, male infertility, and other diseases [10]. Considering the current COVID-19 pandemic across the world due to the severe acute respiratory syndrome coronavirus 2 (SARS-COV-2), it has been reported that angiotensin-converting enzyme-2 (ACE-2) receptors are the target receptors for the SARS COV-2 virus [11, 12] and the nicotine, being the major component in tobacco, it directly impacts the putative receptor of the ACE-2 enzyme making the individual vulnerable and at higher risk for the COVID 19 attack [13].

According to the WHO, there are three million deaths yearly anually due to alcohol consumption, which constitutes around 5.3% of total deaths globally and 5.1% of the global burden of disability-adjusted life years (DALYs) due to alcohol consumption [14]. In India, a study conducted by Girish *et al.* estimates that 13% of the Indian population consumes alcohol, with a higher percentage of males consuming alcohol compared to females [15]. The consumption of alcohol leads to various digestive or cardiovascular diseases, including cancer. Around 900,000 deaths are due to alcohol-related injury across the world [14]. It is estimated that about 336 persons die every day due to alcohol consumption, and 40% of road traffic accidents are related to alcohol intake [16, 17]. Multiple systematic reviews and meta-analyses have shown that with an increase in alcohol consumption, the risk for type 2 diabetes mellitus increases in heavy drinkers [18-20]. Since India is the diabetic capital, there is an urgent need to prevent not only alcohol-associated comorbidities but also alcohol addiction.

The main objective of the current study was to estimate the prevalence of the consumption of legal substances such as alcohol and tobacco in India and discuss various non-pharmacological cost-effective ways (such as yoga) that can restrict the consumption of alcohol and tobacco, thereby preventing people from becoming addicts. There is growing evidence about the positive effects of yoga on the control of type 2 diabetes mellitus [21, 22], stress (one of the precursors/motivation for the use of tobacco and alcohol) [23, 24], and addiction [25]. This helps to control the increasing addiction to legal substance abuse in India.

## Material and Methods

The present study was a part of a larger project - Niyantrita Madhumeha Bharata (NMB), 2017 (Diabetes control in India). This study was a nationally representative door-to-door cross-sectional survey in India. Out of 29 states and 7 union territories in India, 26 states and four union territories were included.

This study was funded by the Ministry of Ayurveda, Yoga, Unani, Siddha and Homeopathy (AYUSH) and the Ministry of Health and Family Welfare, Government of India. The study was approved by the Institutional Ethics Committee of the Indian Yoga Association (IYA), Swami Vivekananda Yoga Anusandhana Samsthana (S-VYASA), Bengaluru (vide Res/IEC-IYA/001 dt 16.12.16). Informed consent was obtained from all the participants during the door-to-door survey. Data were collected from adults above 18 years of age.

**Figure 1:**
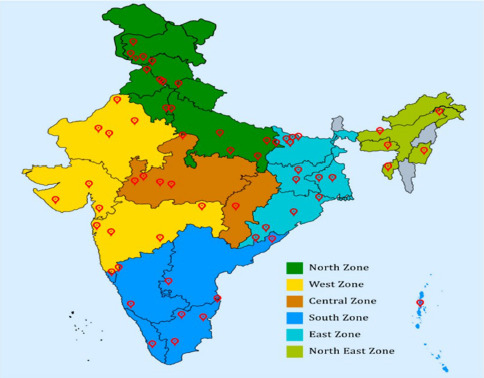
Different zones of India.

The entire methodology of the project has been published in two papers [26, 27]. The whole country was divided into six zones in India, represented in Figure 1. In states with 10 to 30 districts, 2 districts were selected, and from a state with 10 or fewer districts, one district was selected for sampling. The door-to-door survey, which included the basic demographic information of the participants and information about legal substance use (alcohol and tobacco) in India, was used. At the time of carrying out this study, the implications of the COVID pandemic were not foreseeable.

**Figure 2:**
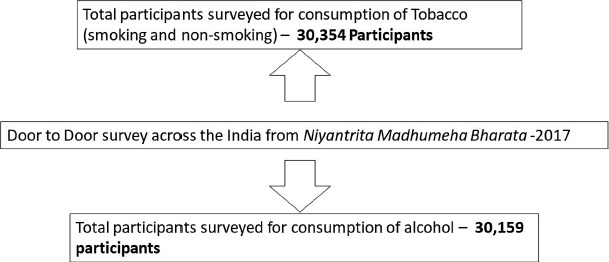
The number of participants from the door-to-door survey.

The data were simultaneously collected from all the regions (Figure 1). Since a large amount of data was obtained, the information was uploaded in the NMB apps and was cross-verified randomly. The hard copies of the data and data centralization were carried out at S-VYASA. The data that was collected for this manuscript is shown in Figure 2. We have excluded the Punjab state from our analysis as the non-response to the questions regarding alcohol and tobacco use were very high and causing statistical errors. The statistical analysis was done using the Statistical Package for Social Sciences (IBM Statistics for windows, SPSS v21.0), and the significance of associations (p-value) were calculated using the chi-square analysis at S-VYSVA, Bengaluru, India.

## Results

The data collected from the door-to-door survey of the NMB 2017 under IYA and S-VYASA University represented in Figure 2 were analyzed. Table 1 shows the prevalence and gender-wise distribution of legal substance abuse (both alcohol and tobacco) in India. We noticed that the prevalence of alcohol abuse (8.7%) was higher than the tobacco abuse (7.9%). When compared among the genders, both alcohol and tobacco consumption was higher among males (15.8% alcohol and 13.1% tobacco) when compared to females (3.2% tobacco and 2.4% alcohol).

**Table 1:** Gender-wise distribution regarding tobacco and alcohol abuse in India.

Gender	Tobacco			P-Value	Alcohol			P-Value
	Abuse	No Abuse	Total		Abuse	No Abuse	Total	
**Male**	1879	12449	14328	<0.001	2252	11.997	14249	<0.001
	**13.1%**	86.9%	100%		15.8%	84.21%	100%	
**Female**	508	15491	15999	<0.001	389	15493	15882	<0.001
	3.2%	96.8%	100%		2.4%	97.6%	100%	
**Transgender**	2	25	27	<0.05	0	28	28	0.00
	8%	92.00%	100%		0%	100%	100%	
**Total**	2389	27965	30354	<0.01	2641	27490	30159	<0.01
	7.9%	92.1%	100%		8.7%	91.3%	100%	

For further analysis, we have divided the tobacco abuse among various states and union territories in India, as shown in Table 2. The highest rate of tobacco abuse was found in Arunachal Pradesh, belonging to the north eastern part of India, and the lowest tobacco abuse was seen in Tripura state.

**Table 2:** Prevalence of tobacco abuse among various states and union territories in India.

State/Union Territory	Gender	No Tobacco Abuse	Tobacco Abuse	Total	P-Value
**Andaman and Nicobar**	Male	108	23	131	
	Female	181	15	196	
	Total	289 (88.4%)	38 (11.6%)	327 (100%)	<0.06
**Andhra Pradesh**	Male	684	29	713	
	Female	595	21	616	
	Total	1279 (94.7%)	50 (5.3%)	1329 (100%)	0.425
**Arunachal Pradesh**	Male	197	60	257	
	Female	240	22	262	
	Total	438 (84.2%)	82 (15.3%)	520 (100%)	<0.001
**Assam**	Male	291	65	356	
	Female	479	15	494	
	Total	770 (90.6%)	80 (9.4%)	850 (100%)	<0.001
**Chandigarh**	Male	67	25	92	
	Female	224	2	226	
	Total	291 (91.5%)	27 (8.5%)	318 (100%)	<0.001
**Chhattisgarh**	Male	210	62	272	
	Female	367	21	388	
	Total	577 (87.4%)	83 (12.6%)	660 (100%)	<0.001
**Delhi**	Male	180	0	180	
	Female	188	3	191	
	Total	368 (99.2%)	3 (0.8%)	371 (100%)	0.091
**Gujarat**	Male	1222	216	1438	
	Female	1218	7	1225	
	Transgender	2	0	2	
Total	2442 (91.6%)	223 (8.4%)	2665 (100%)	<0.001
**Haryana**	Male	73	14	87	
	Female	73	1	74	
	Total	146 (90.7%)	15 (9.3%)	161 (100%)	<0.002
**Jammu and Kashmir**	Male	1176	105	1281	
	Female	1782	31	1813	
	Transgender	8	0	8	
	Total	2996 (95.7%)	136 (4.3%)	3132 (100%)	<0.001
**Jharkhand**	Male	640	109	749	
	Female	838	63	901	
	Total	1485 (85.4%)	136 (14.6%)	1739 (100%)	<0.001
**Karnataka**	Male	2297	206	2503	
	Female	2449	42	2491	
	Transgender	3	0	3	
	Total	5128 (95.3%)	253 (4.7%)	5381 (100%)	<0.001
**Madhya Pradesh**	Male	701	154	855	
	Female	1018	49	1067	
	Transgender	1	0	1	
	Total	1720 (89.4%)	203 (10.6%)	1923 (100%)	<0.001
**Manipur**	Male	222	54	276	
	Female	578	16	594	
	Transgender	1	0	1	
	Total	801 (92.0%)	70 (8.0%)	871 (100%)	<0.001
**Maharashtra**	Male	760	157	917	
	Female	1013	36	1049	
	Total	1773 (89.9%)	193 (10.1%)	1972 (100%)	<0.001
**Meghalaya**	Male	186	7	193	
	Female	304	6	310	
	Total	490(97.4%)	13 (2.6%)	503 (100%)	0.229
**Orissa**	Male	700	55	755	
	Female	737	29	766	
	Total	1437 (94.5%)	84 (5.5%)	1521 (100%)	<0.001
**Pondicherry**	Male	398	31	429	
	Female	438	3	441	
	Total	836 (96.0%)	34 (4.0%)	870(100%)	<0.001
**Rajasthan**	Male	385	207	592	
	Female	339	39	378	
	Transgender	2	0	2	
	Total	726 (74.7%)	246 (25.3%)	972 (100%)	<0.001
**Tamil Nadu**	Male	1055	83	1138	
	Female	1436	24	1460	
	Total	2491 (95.9%)	107 (4.1%)	2598 (100%)	<0.001
**Tripura**	Male	145	1	146	
	Female	104	0	104	
	Total	249 (99.6%)	1 (0.4%)	250 (100%)	0.398
**Uttar Pradesh**	Male	115	7	122	
	Female	56	2	58	
	Total	171(90%)	19 (10%)	190(100%)	0.135
**Uttarakhand**	Male	192	15	207	
	Female	261	2	263	
	Total	453(96.4%)	17 (3.6%)	470(100%)	<0.001
**West Bengal**	Male	224	101	325	
	Female	385	57	442	
	Total	609 (79.4%)	158 (20.6%)	767 (100%)	<0.001
**Total**		27965 (92.1%)	2389 (7.9%)	30354 (100%)	<0.001

**Table 3:** Prevalence of alcohol abuse among various states and union territories in India.

State/Union Territory	Gender	No Alcohol Abuse	Alcohol Abuse	Total	P-Value
**Andaman and Nicobar**	Male	117	14	131	
	Female	195	1	196	
	Total	312 (95.4%)	15 (4.6%)	327 (100%)	<0.001
**Andhra Pradesh**	Male	667	48	715	
	Female	600	19	619	
	Total	1267 (95.0%)	67 (5.0%)	1334 (100%)	<0.002
**Arunachal Pradesh**	Male	152	106	258	
	Female	184	78	262	
	Total	337 (64.7%)	184 (35.3%)	521 (100%)	0.2
**Assam**	Male	260	102	362	
	Female	480	21	501	
	Total	740 (85.7%)	123 (14.3%)	863 (100%)	<0.001
**Chandigarh**	Male	60	33	93	
	Female	223	3	226	
	Total	283 (88.7%)	36 (11.3%)	319 (100%)	<0.001
**Chhattisgarh**	Male	202	73	275	
	Female	377	12	389	
	Total	579 (87.2%)	85 (12.8%)	664 (100%)	<0.001
**Delhi**	Male	190	1	191	
	Female	180	0	181	
	Total	370 (99.7%)	1 (0.3%)	371 (100%)	0.331
**Gujarat**	Male	1323	142	1465	
	Female	1234	13	1247	
	Transgender	2	0	2	
	Total	2559 (94.2%)	155 (5.8%)	2714 (100%)	<0.001
**Haryana**	Male	64	23	87	
	Female	76	0	76	
	Total	140 (85.8%)	23 (14.2%)	163 (100%)	<0.001
**Jammu and Kashmir**	Male	1178	108	1286	
	Female	1811	2	1813	
	Transgender	8	0	8	
	Total	2997 (96.5%)	110 (3.5%)	3107 (100%)	<0.001
**Jharkhand**	Male	626	208	834	
	Female	888	30	918	
	Transgender	9	0	9	
	Total	1523 (86.5%)	238 (13.5%)	1761 (100%)	<0.001
**Karnataka**	Male	2155	349	2504	
	Female	2419	76	2495	
	Transgender	3	0	3	
	Total	4577 (91.5%)	425 (8.5%)	5002 (100%)	<0.001
**Madhya Pradesh**	Male	780	123	903	
	Female	1074	16	1090	
	Transgender	2	0	2	
	Total	1856 (93.0%)	139(7.0%)	1995(100%)	<0.001
**Maharashtra**	Male	135	142	277	
	Female	590	4	594	
	Transgender	1	0	1	
	Total	1796 (91.0%)	177 (9.0%)	1973 (100%)	<0.001
**Manipur**	Male	749	174	923	
	Female	1047	3	1050	
	Total	726 (83.3%)	146 (16.7%)	872 (100%)	<0.001
**Meghalaya**	Male	176	18	194	
	Female	309	1	310	
	Total	485 (96.2%)	19 (3.7%)	504 (100%)	<0.001
**Orissa**	Male	713	54	767	
	Female	698	69	767	
	Total	1411 (92.0%)	123 (8.0%)	1534 (100%)	0.162
**Pondicherry**	Male	440	1	441	
	Female	326	103	429	
	Total	766 (88.0%)	104 (12.0%)	870 (100%)	<0.001
**Rajasthan**	Male	451	135	586	
	Female	348	32	380	
	Transgender	2	0	2	
	Total	801 (82.7%)	167 (17.3%)	969 (100%)	<0.001
**Tamil Nadu**	Male	1452	12	1464	
	Female	944	199	1143	
	Total	2396 (91.9%)	211 (8.1%)	2607 (100%)	<0.001
**Tripura**	Male	102	2	104	
	Female	144	2	146	
	Total	246 (98.4%)	4 (1.6%)	250 (100%)	0.731
**Uttar Pradesh**	Male	60	1	61	
	Female	111	27	138	
	Total	171 (86.0%)	28 (14.0%)	199 (100%)	0.001
**Uttarakhand**	Male	194	13	207	
	Female	261	2	263	
	Total	455 (96.8%)	15 (3.2%)	470 (100%)	0.001
**West Bengal**	Male	288	39	327	
	Female	437	7	444	
	Total	725 (94.0%)	15 (6.0%)	771 (100%)	<0.001
Total		27490 (91.3%)	2641 (8.7%)	30159 (100%)	<0.001

As with tobacco abuse, we have divided the prevalence of alcohol abuse among various states and union territories in India, as shown in Table 3. The highest percentage of alcohol abuse was found in Arunachal Pradesh, and the least alcohol abuse was seen in the Tripura state. The prevalence pattern of alcohol abuse is similar to the tobacco abuse pattern. The weighted percentages of the individual states and union territories were included in Table 4.

**Table 4:** The weighted percentages of the individual states for subjects that abuse tobacco and alcohol included in the study.

State/Union Territory	Weighted Percentage	
	Tobacco	Alcohol
**Andaman and Nicobar**	1.08%	1.08%
**Andhra Pradesh**	4.38%	4.44%
**Arunachal Pradesh**	1.71%	1.78%
**Assam**	2.80%	2.86%
**Chandigarh**	1.05%	1.06%
**Chhattisgarh**	2.17%	2.20%
**Delhi**	1.22%	1.23%
**Gujarat**	8.78%	9.00%
**Haryana**	0.53%	0.54%
**Jammu and Kashmir**	10.32%	10.30%
**Jharkhand**	5.73%	5.84%
**Karnataka**	17.73%	16.59%
**Madhya Pradesh**	6.34%	6.61%
**Manipur**	2.87%	2.89%
**Maharashtra**	6.50%	6.54%
**Meghalaya**	1.68%	1.67%
**Orissa**	5.01%	5.08%
**Pondicherry**	2.87%	2.88%
**Rajasthan**	3.20%	3.21%
**Tamil Nadu**	8.56%	8.64%
**Tripura**	0.82%	0.83%
**Uttar Pradesh**	0.62%	0.66%
**Uttarakhand**	1.55%	1.56%
**West Bengal**	2.53%	2.56%
**Total**	100%	100%

**Table 5:** Summary of the AYUSH Diabetes Yoga Protocol [25].

S. No	Name of the Practice	Duration (in min)
1.	**Starting Prayer:** Asatoma Sat Gamaya Tataso Maa jyotir - gataya Mrtyor-Maa Amrtam gamaya Om Shaantih Shaantih Shaantih Meaning: From ignorance, lead me to truth; From darkness, lead me to light; From death, lead me to immortality; ‘Om peace, peace, peace.	2
2.	**Loosening Exercises** (Preparatory ^Sukshma Vyayamas^ and ^Shithililarna^ Practices): **1.** ***Urdhavahastashvasan*** (Upward Tree Position) (Hand Stretch Breathing 3 rounds at 90 degrees, 135 degrees and 180 degrees each) **2.** ***Kati-Shakti Vikasaka*** (3 rounds) **a.** Forward and Backward Bending; **b.** Twisting. **3.** ***Sarvangapushti*** (3 rounds clockwise, 3 rounds anticlockwise)	6
3.	**Surya Namaskara (SN)** (Sun Salutation) **a.** 10-step fast ^Suryanamaskara^ (Fast Sun Salutation) 6 rounds; **b.** 12-step slow ^Suryanamaskara^ (Slow Sun Salutation) 1 round. Modified version Chair SN: 7 rounds	9
4.	***Asanas*** **(Pose/Posture)** (^1 min per Asana^) **1. Standing Position** (^1 min per Asana^) ^Trikonasana^ (extended triangle pose), ^Parvritta Trikonasana^ (revolved triangle pose), ^Prasarita Padhastasana^ (Wide-Legged Forward Bend) **2. Supine Position** ^Jatara Parivartanasana^ (Master Revolved Abdomen Pose), ^Pawanamuktasana^ (Wind-Relieving Pose), ^Viparitakarani^ (Upside-Down pose) **3. Prone Position** ^Bhujangasana^ (Cobra Pose), ^Dhaurasana^ (Bow Pose) followed by ^Pawanamuktasana^ (Wind-Relieving Pose) **4. Sitting Position** ^Mandukasana^ (Frog Pose), ^Vakrasana / Ardhamatsayendrasana^ (Half Spinal Twist Pose), ^Paschimatanasana^ (Seated Forward Bend), ^Ardha Ushtrasana^ (Half Camel Pose); At the end, relaxation with abdominal breathing in supine position (^vishranti^), 10-15 rounds (2 minutes)	15
5.	**Kriya** (Outward Physical Manifestation) **a.** ^Agnisara^ (Abdomen Churning): 1 minute, **b.** ^Kapalabhati^ (Skull Shining Breathing Technique) (^60 breaths per minute for 1 minute followed by rest for 1 minute^)	3
6.	***Pranayama*** (Breathing Techniques) **a.** ^Nadishuddhi^ (Alternate Nostril Breathing) [^for 6 minutes, with antarkumbhak^ (Internal Breath Retention) ^and jalandhar bandha^ (Chin Lock) ^for 2 seconds^] **b.** ^Bhramari^ (Humming Bee Breathing): ^3 minutes^	9
7.	Meditation (for stress, for deep relaxation and silencing of mind) Cyclic Meditation	15
8.	**Closing Prayer:** Sarvebhavantu Sukhina Sarve Santu Nirāmayaah Sarve Bhadrani Paśyantu Maa KaScid-Duhkha-Bhag-Bhavet Om Shaantih Shaantih Shaantih **Meaning:** Let all be happy, free from diseases. Let all align with reality, let no-one suffer from miseries. ‘Om peace, peace, peace.	1
	Total duration	60

## Discussion

From the data of 30,354 participants that abuse tobacco and 30,159 participants that abuse alcohol, it was seen that 7.9% and 8.7% of people abuse tobacco and alcohol, respectively. This is the nationally representative population. In a study conducted by Prakash *et al.*, it was noted that among 35,102 men aged above 45 years, the prevalence of tobacco use was around 15%, which is close to our study where a similar percentage of 13.1% of tobacco abuse was seen in men. The slight difference might be due to the inclusion of people aged 18 years or above in our sample [28]. It is also noted that more than 50% of people who consume alcohol are also tobacco abusers, which was concluded in the same study [28]. Due to the lack of awareness among the people in rural areas, a higher prevalence of tobacco and alcohol consumption of more than 30% is seen especially in the older adults across various places in India [29, 30]. It was also noticed in various studies that tobacco and alcohol constitute a major risk for non-communicable diseases such as cardiovascular diseases, cancer, and others [31, 32]. Also, there is no comprehensive study on the usage of substances by adolescents. In a large sample study done by Jaisoorya *et al.*, the prevalence of psychological distress was reported to be around 34.8% among college-going students and adolescents. This fact seems to have negative outcomes, especially in the case of substances that lead to addictions gradually [33]. Therefore, the importance of preventing the usage of substance abuse in adolescents is highlighted.

It is generally perceived that there is a lack of strict regulation about tobacco and alcohol abuse because of the apparent link with Government revenue generated for the country by the tobacco and alcohol industry annually. For example, the tax revenue in 2019 generated from smoking cigarettes alone is 348.34 billion Indian rupees, which is 15% higher than the 2018 fiscal year, pointing to the increase in the consumption of tobacco annually [34]. Similar estimates of excise revenues from the alcohol industry are approaching 10.4 trillion Indian rupees in 2019-20. By 2023, it is estimated the sales will increase [35]. In contrast, the health care GDP of India has remained at one percent of the last ten years since 2009, although the GDP per-capita of Indians has doubled between 2009-10 and 2017-18. However, the GDP of developed countries such as the United States of America is around 18% [36]. Considering the statistics, the need of cost-effective solutions towards substance abuse is required at various levels of age groups to increase the health of the country, especially in times of the COVID19 pandemic.

One of the cost-effective solutions that should be considered for implementation in order to reduce legal substance abuse and its associated comorbidities is yoga, especially the Diabetic Yoga Protocol (DYP) developed by the Ministry of AYUSH by a 16-member committee across the country (Figure 5) [37, 38]. The DYP protocol is a 60-minute session with a regular follow-up that can facilitate both the release of stress caused by the closure of liquor and wine shops in the country during the current lockdown and also prevent the conversion of prediabetes to diabetes [21, 23, 25]. This is highly required, especially in the northern states of India, such as Arunachal Pradesh, due to the high usage of legal substances.

### School and college-based mandatory yoga programs to control psychosocial stress

The adolescent and college-going students display psychosocial and academic stress [39], which is higher than most countries partly due to the population of the country [40]. The increasing propensity towards substance abuse such as alcohol and tobacco [35, 39] also renders tobacco consumers and other addicts that are more vulnerable to diabetes and COVID-19 infection [41]. To reduce such stress among adolescents and college-going adults, a mandatory three-day yoga programme per week needs to be implemented [37]. This yoga protocol has been shown to reduce stress and control diabetes in the nation-wide study that took place in India [42, 43]. The early implementation of such protocols among teenagers that go to school coupled with awareness about the harmful effects of smoking and alcohol abuse can result in substantial reduction and prevention of addiction in the near future [44].

### Mindfulness-based DYP into the wellness centers of Ayushmann Bharat

In February 2018, the Indian government had launched a universal health coverage program known as Ayushmann Bharat to control non-communicable diseases. As a part of the program, 150,000 public peripheral health centers focused on health and wellness agenda are being operationalized for delivering comprehensive primary health care by the end of 2022 [45, 46]. As a part of these centers, mindfulness-based DYP must be introduced into these wellness centers to reach out to the public, which will be a cost-effective non-pharmacologic way to reduce substance abuse in India. Several randomized controlled trials on the effect of yoga [47, 48] and mindfulness [25] on substance abuse have shown positive results towards the cessation of substance abuse over time. The introduction of the mindfulness-based DYP into the wellness centers will prevent substance abuse at the initial stages and decrease the global disease burden.

The sample used in this study was collected across the country; however, the sample is not representative of each individual state, and a convenient sample size was taken from each state. In some states, the non-response rate for the questions about tobacco and alcohol abuse was high, and states such as Punjab were excluded from the data collection process to prevent statistical errors. There might have been reporting bias in the statements of individuals who have abused tobacco and alcohol only once. This paper did not help us give the right percentages of tobacco and alcohol abuse in the transgender population as this population sample is insufficient.

## Conclusion

From this current study, it is estimated that around 8.7% of alcohol and 7.9% of tobacco users exist in India, with the highest rate of tobacco abuse in Arunachal Pradesh and the lowest in Tripura (for both types of substance abuse). To prevent the disease burden from legal substances abuse, a cost-effective non-pharmacological approach (considering the GDP of India for health) is required. Such approaches include a school/college-based mandatory yoga program to control psychosocial stress in adolescents, including the installation of DYP into the wellness centers of Ayushmann Bharat for the general population to prevent legal substance abuse and decrease the disease burden on the country.

## Acknowledgments

This study was funded by Ministry of Health and Family Welfare, Ministry of AYUSH, Government of India routed through the Central Council for Research in Yoga and Naturopathy (CCRYN) and implemented by the Indian Yoga Association (F. No. 16-63/2016-17/CCRYN/RES/Y&amp;D/MCT/Dated: 15.12.2016).

## Conflict of Interest

The authors declare that there is no conflict of interest.
